# Cattle farmer psychosocial profiles and their association with control strategies for bovine viral diarrhea

**DOI:** 10.3168/jds.2021-21386

**Published:** 2022-04

**Authors:** N.S. Prosser, M.J. Green, E. Ferguson, M.J. Tildesley, E.M. Hill, M.J. Keeling, J. Kaler

**Affiliations:** 1 School of Veterinary Medicine and Science, University of Nottingham, Sutton Bonington Campus, Leicestershire, LE12 5RD, United Kingdom; 2 School of Psychology, University Park, University of Nottingham, Nottingham, NG7 2RD, United Kingdom; 3 Zeeman Institute for Systems Biology & Infectious Disease Epidemiology Research, School of Life Sciences and Mathematics Institute, University of Warwick, Coventry, CV4 7AL, United Kingdom; 4 Joint UNIversities Pandemic and Epidemiological Research (JUNIPER; https://maths.org/juniper/)

**Keywords:** bovine viral diarrhea, COM-B, farmer behavior, psychological proximity, psychosocial profiling

## Abstract

Bovine viral diarrhea (BVD) is endemic in the United Kingdom and causes major economic losses. Control is largely voluntary for individual farmers and is likely to be influenced by psychosocial factors, such as altruism, trust, and psychological proximity (feeling close) to relevant “others,” such as farmers, veterinarians, the government, and their cows. These psychosocial factors (factors with both psychological and social aspects) are important determinants of how people make decisions related to their own health, many of which have not been studied in the context of infectious disease control by farmers. Farmer psychosocial profiles were investigated using multiple validated measures in an observational survey of 475 UK cattle farmers using the capability, opportunity, motivation-behavior (COM-B) framework. Farmers were clustered by their BVD control practices using latent class analysis. Farmers were split into 5 BVD control behavior classes, which were tested for associations with the psychosocial and COM-B factors using multinomial logistic regression, with doing nothing as the baseline class. Farmers who were controlling disease both for themselves and others were more likely to do something to control BVD (e.g., test, vaccinate). Farmers who did not trust other farmers, had high psychological capability (knowledge and understanding of how to control disease), and had high physical opportunity (time and money to control disease) were more likely to have a closed, separate herd and test. Farmers who did not trust other farmers were also more likely to undertake many prevention strategies with an open herd. Farmers with high automatic motivation (habits and emotions) and reflective motivation (decisions and goals) were more likely to vaccinate and test, alone or in combination with other controls. Farmers with high psychological proximity (feeling of closeness) to their veterinarian were more likely to undertake many prevention strategies in an open herd. Farmers with high psychological proximity to dairy farmers and low psychological proximity to beef farmers were more likely to keep their herd closed and separate and test or vaccinate and test. Farmers who had a lot of trust in other farmers and invested in them, rather than keeping everything for themselves, were more likely to be careful introducing new stock and test. In conclusion, farmer psychosocial factors were associated with strategies for BVD control in UK cattle farmers. Psychological proximity to veterinarians was a novel factor associated with proactive BVD control and was more important than the more extensively investigated trust. These findings highlight the importance of a close veterinarian-farmer relationship and are important for promoting effective BVD control by farmers, which has implications for successful nationwide BVD control and eradication schemes.

## INTRODUCTION

Bovine viral diarrhea (**BVD**) is endemic in the United Kingdom and causes major economic losses that result from poor growth rates and pneumonia, reduced milk production, reduced fertility, fatalities from mucosal disease, and increased susceptibility to other diseases (Houe, 1999; Weldegebriel et al., 2009). Bovine viral diarrhea transmission occurs primarily through persistently infected (**PI**) cattle, which are created when the dam becomes infected early in pregnancy (McClurkin et al., 1984). These calves are immunotolerant to the BVD virus (Peterhans et al., 2003) and shed virus their entire life. Other cattle are transiently infected and act as a weaker source of infection, being infectious for a much shorter period.

There are many strategies farmers can adopt to control BVD, ranging from measures to prevent the introduction and transmission of infectious diseases in general to the BVD specific measures of testing, culling PI cattle, and vaccination. The decisions farmers make within their own herd have implications for national disease control, with frequent movements between cattle holdings and markets in the United Kingdom (Vernon, 2011) and the potential for local disease transmission between neighboring herds (Abernethy et al., 2011; Graham et al., 2016). There is a potential incentive to free-ride and rely on other people to control disease (Bauch and Earn, 2004). Where national BVD prevalence is high, maintaining freedom from disease in a herd is more costly (Gunn et al., 2005). Bovine viral diarrhea control is voluntary in England and Wales, and farmers can engage with voluntary schemes [BVDFree (BVDFree, 2021) and Gwaredu BVD (Gwaredu BVD, 2019)]. In contrast, BVD testing is mandatory in breeding herds in Scotland and Northern Ireland, with movement restrictions on PI cattle to incentivize control.

The undertaking of any given behavior is influenced by a person having sufficient capability (e.g., physical ability, knowledge, and understanding), opportunity (e.g., physical resources and support from others), and motivation (e.g., both reflective decision making and automatic habits and emotions; Michie et al., 2011, 2014). The capability, opportunity, motivation-behavior (**COM-B**) framework of behavior change captures these interrelated attributes, encompasses existing frameworks for behavior in health settings, and has been applied to farmer and veterinary behavior (Michie et al., 2011; Hardefeldt et al., 2018; Carroll and Groarke, 2019). The COM-B framework has traditionally been used to study predictors of individual behavior change. However, infection control, be it via vaccination or behavioral controls, requires an appreciation of the dynamic relationship with people. For example, vaccination carries a personal cost, but benefits both the person vaccinated and those not vaccinated. Thus, the nonvaccinated can pay no cost and free-ride on others' decisions to vaccinate (Bauch and Earn, 2004; Böhm et al., 2016). Therefore, to fully understand infection control decisions it is necessary to include assessment of key mechanisms underlying cooperation: generosity, altruism, trust, fairness, and proximity (Fehr and Fischbacher, 2003; Nowak, 2006; Rand and Nowak, 2013; Bradley et al., 2018; Dimick et al., 2018). These psychosocial factors (factors with both psychological and social aspects) come from a separate theoretical literature to the COM-B framework and are more intrinsic to a person's nature than the COM-B factors.

People with altruistic (defined here as seeking to benefit others at a personal cost; West et al., 2007; Bshary and Bergmüller, 2008; Pfattheicher et al., 2022) and prosocial (aim for equality) preferences tend to make health decisions that benefit others. In contrast, people with more selfish or proself (seek to benefit self) preferences make decisions that benefit themselves. For example, prosocial individuals are more likely to get vaccinated, unlike proself people who are more likely to rely on herd immunity (Böhm et al., 2016). People may also show reactive reluctant altruism, behaving in a way that benefits others but only because they do not trust others to help, which, for example, is important in blood donation behavior (Ferguson et al., 2012; Ferguson, 2022). Behaviorally, generosity and altruism can be investigated using dictator games where a decision maker chooses how to split an endowment between themselves and another person (Forsythe et al., 1994). The dictator game can be modified with different recipients and different contexts for the original endowment that is to be split (e.g., it could have been earned, a gift, or lottery winnings) to investigate how altruistic the decision maker is (Engel, 2011). Altruism can also be investigated using the social value orientation (**SVO**) slider measure (Murphy et al., 2011) where participants make 6 dictator game decisions from a series of set responses. Their responses are used to calculate a score of how altruistic they are which can used to categorize participants as competitive (<−12.04, maximizes the difference in benefit to self versus benefit to other), individualistic (−12.04 to 22.45, maximizes benefit to self), prosocial (22.45 to 57.15, maximizes joint gain or minimizes inequality), or altruistic (>57.15, maximizes benefit to other; Murphy et al., 2011).

People are generally more likely to help those they feel close to or are psychologically proximal to (Cialdini et al., 1997). High psychological proximity is associated with increased uptake of behavior to protect other people's health (Tu et al., 2021) and increased support for others to change their behavior (Bobak and Raupach, 2018). Psychological proximity can be measured using the inclusion of other in self (**IOS**) scale (Aron et al., 1992), where participants select a pair of overlapping circles from a scale of increasingly overlapping circles that best represents how close they feel to a specific “other” in question. Trust that others will control infectious diseases is another important factor and promotes cooperation with prosocial disease control behavior such as BVD control schemes (Heffernan et al., 2016; Pletzer et al., 2018). Cattle farmers in the United Kingdom generally have high trust in veterinary advice and low trust in government policy. They are more likely to follow trusted veterinary advice than want to cooperate with government recommendations (Brennan and Christley, 2013; Fisher, 2013; Bard et al., 2019). Behaviorally, trust and trustworthiness can be investigated with investment games (also known as trust games; Berg et al., 1995). In these games the investor chooses how much of an endowment to invest in another unknown person. The amount invested is multiplied (usually tripled) and the investee chooses how much of the multiplied investment to return to the investor. Thus, increasing the amount invested indexes how trusting the investor is that the investee will act in a trustworthy manner and return a fair amount on the investment. Expectations of trust in others can be gauged by asking investors to estimate how much they believe an investee will return. Thus, the trust game also taps concepts of cooperation, and reciprocity (Ferguson et al., 2020). Modifications can also be made to this game for the particular context to be studied.

In this paper we investigate psychosocial profiles of UK cattle farmers, many of which have not been investigated in farmers to date, to evaluate how individual profiles and factors from the COM-B framework of behavior change, are associated with the farmers' strategy to control BVD in their herd.

## MATERIALS AND METHODS

Ethical approval was obtained from the University of Nottingham Research Ethics Committee for both the focus groups and the survey before commencement of the study (reference number: 2789 190711, granted: June 22, 2019).

### Focus Group Design, Recruitment, and Analysis

A series of focus groups (n = 4) was conducted to inform the development of a farmer survey of psychosocial characteristics and infectious disease control. A focus group guide was designed to investigate altruism, trust, psychological proximity, and the COM-B framework of behavior change (Michie et al., 2011) in the context of BVD control in a 1-h discussion. The facilitator used the questions and prompts in the focus guide to start the discussion and ensure that the farmers discussed all areas of interest, but otherwise allowed the farmers to talk freely. During the focus group, participants were provided with a printout of the IOS scale, a measure of psychological proximity (Aron et al., 1992; Mashek et al., 2007), as a discussion aid (Supplemental Figure S1; https://rdmc.nottingham.ac.uk/handle/internal/9483). The question guide was modified slightly between the first and subsequent focus groups to improve probing on other-regarding preferences (e.g., prosociality, altruism). The same facilitator conducted all focus groups.

Focus groups were conducted in February 2020 in 3 geographical areas (Nottinghamshire, Somerset, and Yorkshire), with 3 to 8 farmers per group. All farmers had a minimum of 100 dairy cows, and each group contained both farmers who were part of the national BVD eradication program (BVDFree England) and those who were not. Three of the 4 focus groups also contained both farmers who had regular routine visits from their veterinarian and those who did not. Farmers were recruited as a convenience sample from herds associated with the University of Nottingham (2 focus groups) and 2 veterinary practices (2 focus groups). The focus groups took place at the Centre for Dairy Science Innovation at the University of Nottingham or at veterinary practices and were recorded and transcribed by an external agency (Penguin Transcription). Transcripts were checked once against the audio file by the author (NP). Theoretical thematic analysis (Braun and Clarke, 2006) of all transcripts was conducted to identify and explore the psychosocial constructs that should be included on the broader farmer survey.

### Survey Design

A survey was designed to cover a wide spectrum of relevant other-regarding preferences. These included altruism (West et al., 2007; Bshary and Bergmüller, 2008; Pfattheicher et al., 2022), reactive reluctant altruism (helping because of lack of trust that others will help; Ferguson et al., 2012; Ferguson, 2022), and trust and distrust (McKnight and Chervany, 2001). Altruism was assessed using the SVO slider measure questions in which farmers chose to allocate money between themselves and another unknown farmer (Murphy et al., 2011). This task was incentivized; 10 farmers were selected at random to receive a financial reward for one of their decisions, paid to themselves and another random survey respondent; payment based on one decision is commonly used for this type of economic decision and has the advantage of reducing hedging (Charness et al., 2016). Altruism was also investigated using a dictator game where farmers could share £700 ($877.80) hypothetical lottery money between themselves, an unknown farmer, a known farmer, an unknown veterinarian, a known veterinarian, and a stranger. Hypothetical pay does not alter dictator game decisions (Engel, 2011). Trust was investigated using an investment game (Berg et al., 1995) where £50 hypothetical lottery winnings could be invested in another unknown farmer. Investments were tripled and the farmers were asked how much they would expect the other farmer to return. Altruism, reluctant altruism, trust, and distrust were also investigated by Likert-scale questions created by the authors investigating trust and distrust in farmers, veterinarians, government, strangers, the National Farmers' Union, and the farming press (section 4, question 4 in Supplemental File S1; https://rdmc.nottingham.ac.uk/handle/internal/9483).

We assessed factors associated with other-regarding preferences such as psychological proximity (Aron et al., 1992; Mashek et al., 2007), general motivation for behavior using the COM-B framework (Michie et al., 2014), and anxiety about BVD. Psychological proximity with farmers, veterinarians, the government, the National Farmers' Union, and their cows was investigated using the IOS scale (Aron et al., 1992; Mashek et al., 2007). Each set of increasingly overlapping circles was given a score of 1 to 7, with higher numbers for increased overlap which represented increased psychological proximity (Supplemental Figure S1). The COM-B questions were based on a published question guide (Michie et al., 2014), with additional relevant questions from other published uses of the guide (Barker et al., 2016; Taylor et al., 2016; Bobak and Raupach, 2018). The COM-B questions were used to explore psychological capability (knowledge and understanding), physical opportunity (time and money), social opportunity (support from others), automatic motivation (habits and emotions), and reflective motivation (plans and goals). Physical capability (ability) was not considered relevant so was not investigated in the survey. We considered that a lack of physical capability would preclude farmers from keeping cattle; therefore, it was not applicable to this group of people. Anxiety about BVD was assessed on a 5-point scale of how anxious farmers felt about a BVD breakdown on their farm. Farmers were also asked questions to gather demographic information and to capture their current implementation of BVD control strategies.

The survey was tested by members of the research group and in a pilot study conducted using a convenience sample of 8 cattle farmers. The final survey in full is provided in Supplemental File S1.

### Survey Dissemination

The survey was open from July 13 to October 5, 2020. The survey link was emailed to 10,560 British dairy and English beef levy payers by the Agriculture and Horticulture Development Board, and various cattle interest organizations also promoted the survey via magazine articles, e-newsletters, social media posts, website posts, and emails to UK cattle farmers. A hardcopy was also posted to a random sample of 2,000 of the dairy and 2,000 of the beef levy payers. The random selection for the farmers who were rewarded with a payment based on their SVO decisions was conducted in R statistical software (v3.6.2; R Core Team, 2019).

### Data Entry and Analysis

Data entry for the postal surveys was conducted by an outside agency (Wyman Dillon Ltd.), except for the final 8 late returns which were conducted by the author (NP). All data analysis was conducted using R statistical software (v3.6.2; R Core Team, 2019) and each analysis used only the complete responses for the relevant survey questions.

### Factor Analyses

To identify latent groupings and reduce the number of variables for analysis, factor analysis (Thurstone, 1947) was conducted on 32 Likert-scale items regarding reluctant altruism, altruistic or proself preferences, trust, and distrust. The factor analysis was conducted using the psych package (v2.0.8) with maximum likelihood and oblimin rotation (Revelle, 2020). Likert-scale questions were converted to numeric (1–5 for ascending strength of agreement). The number of factors was chosen based on parallel analysis, fit statistics (Tucker-Lewis index ≥0.9 and root mean squared error of approximation ≤0.06; Hu and Bentler, 1999), and a minimum of 2 variables loaded to each factor. Loadings ≥0.3 were considered to define a factor. Items that were cross-loaded on 2 factors were retained in the model if their omission did not change the model fit. Factor scores for each respondent were calculated from every statement in the factor analysis, weighted by its loading (regardless of size of loading). Cronbach's α was calculated for each factor to assess internal reliability. If Cronbach's α was <0.7 and could be improved with omission of an item, the item was omitted.

The COM-B items were grouped by their overarching factor (psychological capability, physical opportunity, social opportunity, automatic motivation, and reflective motivation). The Likert-scale responses were also converted to a numeric response (1–5 for ascending strength of agreement). Factor scores were created by taking the mean score of the items in that factor, with an item's scale inverted if necessary (Supplemental Table S1; https://rdmc.nottingham.ac.uk/handle/internal/9483).

### Latent Class Analysis

Two latent class analyses were conducted to identify clusters of farmers from their responses:

(1) Three items from the economic games were rescaled to proportions for comparative purposes: the proportion of £50 that each farmer invested, the proportion of tripled investment each farmer expected to be returned in the investment game, and the proportion of £700 each farmer gave away in the dictator game.
(2) The BVD control behaviors of the farmers: buy only from BVD-free herds, closed herd, disinfection for people entering the farm, separation from neighboring stock, isolate or test new cattle, vaccinate, blood or tissue test, milk test, cull PI, and isolate sick animals.

Latent class analysis was conducted using the mclust (v5.4.6) package (Fraley and Raftery, 2002; Scrucca et al., 2016). Models were selected as the spherical or diagonal mixture model with the highest Bayesian information criterion (**BIC**) where at least 5% of farmers were in each class and there was good delineation of the classes (normalized entropy >0.7).

### Multinomial Logistic Regression

To investigate associations between farmer BVD control behaviors and psychosocial factors, the BVD latent classes were evaluated as a multinomial outcome in a logistic regression model with explanatory covariates: economic games latent class, altruism and trust factors, psychological proximity to others, each COM-B factor (psychological capability, physical opportunity, social opportunity, automatic motivation, and reflective motivation), and anxiety about a BVD breakdown. The models were built with the nnet (v7.3.12) package (Venables and Ripley, 2002).

Each independent variable was initially tested in a univariable model and initially all significant variables (*P* < 0.05) were selected to test in a multivariable model. Terms that were not significant in the multilevel model were removed from the model and all terms were retested in the final model. Spearman rank correlations were calculated for all variables considered for inclusion in the multivariable model. Variables that were correlated (≥0.3) with a variable in the final model were tested in the model in place of their correlated variable. Models where correlated terms were significant when substituted in the final model are presented as alternative models. Demographic data were not included in the multivariable model because these were all categorical and there were insufficient respondents to avoid small and empty contingency table cells in the model. However, each demographic variable was tested in the final model to check for confounding. The United Kingdom was split into northern (Scotland, Northern Ireland, and English counties north of and including Cheshire, Derbyshire, Nottinghamshire, and Lincolnshire) and southern (Wales and all remaining English counties) areas to test in the model. Additionally, the southwest (Gloucestershire, Wiltshire, Dorset, and more southwesterly counties) were separated out and the variable retested in the model (north, south, southwest). Model fit was evaluated by predicting BVD behavior class from both the full and 10 × 10-fold cross-validated models and comparing the proportion of times the correct class was predicted, and with a Hosmer-Lemeshow goodness-of-fit test (Fagerland and Hosmer, 2012).

Conditional autoregressive models (Lee, 2013) were created to investigate potential spatial confounding of the psychosocial factors at the county level. No confounding was identified and the methods and results for these are presented in Supplemental File S2 (https://rdmc.nottingham.ac.uk/handle/internal/9483).

## RESULTS

### Descriptive Statistics of Survey Respondents

A total of 291 online survey responses were received from July 16 to October 5, 2020, which was 50.4% (/577) of started surveys. A further 184 surveys were returned by post (response rate of 4.6%), making a total of 475 survey responses. The mean pay-out to the 20 prize-winning farmers was £74.25 (range £15–£100). Most respondents were in England (73%), with 14% in Scotland, 7% in Wales, and 2% in Northern Ireland. Most farmers were in their 50s and 60s (30% and 25%, respectively), with 7% under 30, 10% in their 30s, 16% in their 40s, and 11% over 70. Seventy-six percent of farmers had beef cattle, with a median of 70 animals over 6 mo old (range 0–850), and 39% had dairy cattle, with a median of 180 adult cows (range 2–1,309).

### Farmer SVO and Levels of Trust and Distrust

Most farmers were categorized by their SVO slider measure responses as prosocial (75.4%), 16.6% were individualistic, and very few were altruistic (1.3%) or competitive (0.4%).

Veterinarians were the most trusted group, least distrusted group, and farmers also felt the most respected by them. Eighty-one percent of farmers trusted their veterinarian compared with the National Farmers' Union (57%), dairy farmers (47%), beef farmers (38%), and governmental organizations (19%). More farmers agreed that they felt respected by their veterinarian (85%), with 67% agreeing that they felt respected by the veterinary profession, whereas 49% and 10% felt respected by the National Farmers' Union and government, respectively. Similarly, only 31% of farmers thought it was better to be careful before you trust veterinarians, rising to 56% for farmers, 63% for government, and 76% for strangers. Only 17% trusted farmers they met for the first time.

Trust in veterinarians was even higher when it was specifically for veterinary services and infectious disease control compared with general trust: 90% trusted their veterinarian's advice, 90% agreed that their veterinarian would always tell them the truth even if it was not what they wanted to hear, and 80% agreed that farmers received high-quality advice from the veterinary profession. There was less trust in other stakeholders, with 43% agreeing that infectious disease information in the farming press was trustworthy, 35% and 30% trusting their neighbors and other farmers nationally, respectively, to control infectious diseases, and only 16% of farmers trusting governmental judgments about disease control.

A 7-factor solution gave the best fit in a factor analysis of 436 complete responses to the other-regarding preferences, reluctant altruism, trust, and distrust Likert-scale measures (Tucker-Lewis index = 0.88, root mean squared error of approximation = 0.06; Table 1). “I trust other farmers I meet for the first time” loaded on both “trust in farmers” and “general distrust” factors (0.41 and −0.35, respectively) and was retained in the model because its omission did not alter the model. Factor loadings are presented in Table 1. Cronbach's α was >0.7 for all factors except “general distrust”; however, this was not improved by omission of any of its items (Table 1).

**Table 1:** The items loaded (>0.3) onto each of the 7 factors from a factor analysis of 436 complete responses to Likert-scale items on reluctant altruism, altruistic or proself preferences, trust, and distrust in a survey of UK cattle farmers

Factor^1^	Item^2^	Loading
Trust in National Farmers' Union (α = 0.91)	I feel respected by the National Farmers' Union.	0.99
	I trust the National Farmers' Union.	0.82
Reluctant altruism (α = 0.87)	I vaccinate my cows because I cannot trust other farmers to vaccinate theirs.	0.94
	I vaccinate my cows to protect my herd and those around me, because other farmers will not vaccinate.	0.80
Trust in veterinarians (α = 0.87)	I trust my vet's advice about infectious disease control in my herd.	0.88
	My vet would always tell me the truth even if it was not what I wanted to hear.	0.81
	I trust vets.	0.61
	Farmers receive high quality veterinary advice from the veterinary profession.	0.59
	I feel respected by my vet.	0.58
	I feel respected by the veterinary profession.	0.49
Trust in farmers (α = 0.79)	I trust my neighbors to be controlling infectious diseases in their herds.	0.78
	I trust other farmers nationally to be controlling infectious diseases in their herd.	0.70
	I trust beef farmers.	0.65
	I trust dairy farmers.	0.50
	I trust other farmers I meet for the first time.	0.41
Controlling disease for self and others (α = 0.81)	I control infectious disease because I take pride in having a healthy herd.	0.86
	I control infectious disease to protect my reputation for having healthy cattle.	0.70
	Controlling infectious disease in the UK will have benefits for every farmer.	0.69
	I control infectious disease to do my bit for national disease control.	0.68
	I control infectious disease in my cattle to protect my own herd.	0.51
	I control infectious disease in my cattle to protect other farmers' herds.	0.43
Trust in government (α = 0.76)	I trust governmental judgments about how to control infectious diseases in cattle.	0.78
	I feel respected by the government.	0.76
	I trust governmental organizations.	0.65
	When dealing with the government it is better to be careful before you trust them.	−0.44
General distrust (α = 0.57)	When dealing with strangers it is better to be careful before you trust them.	0.48
	When dealing with farmers it is better to be careful before you trust them.	0.47
	When dealing with vets it is better to be careful before you trust them.	0.40
	In general, one can trust people.	−0.43
Items that did not load on any factor	I only control infectious disease when other farmers are also taking steps to control disease.	—
	I control infectious disease to stay ahead of other farmers.	—
	Infectious disease information in the farming press is trustworthy.	—

1 α = Cronbach's α.

2 vet = veterinarian.

### Farmer Behaviors in the Economic Games

Seventy-four percent of farmers made an investment in the trust game. The mean proportion invested by the investors was 0.53 of £50 (range 0.02–1.00) and the mean proportion of the tripled investment that the investors expected the other farmer to return was 0.42 (range 0.00–1.00).

Forty-seven percent of farmers gave some money in the dictator game, with both the total generosity and its distribution between the recipients varying by farmer. For the farmers who gave money, the mean proportion given away was 0.39 of £700 (range 0.01–1.00). The farmers were most generous to neighboring farmers with a mean gift of 0.16 of the lottery money, the local veterinarian received 0.12, the unknown farmer 0.05, the stranger 0.04, and the unknown veterinarian 0.02.

**Figure 1:**
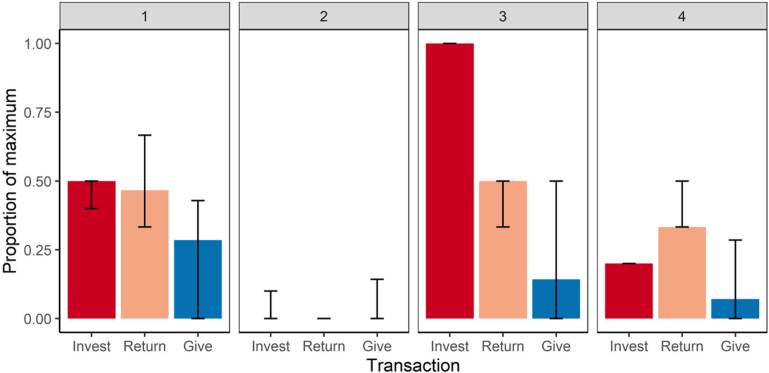
The median responses and interquartile range of the 4 latent classes of the behaviors of 417 farmers in the economic games. Invest = proportion of £50 ($62.70) invested in an unknown farmer in the investment game; return = proportion of the tripled investment expected back from the unknown farmer; give = proportion of £700 given away to others in the dictator game. The latent classes are as follows: 1 = generous self-oriented mutual benefit: the most generous class that invests some and expects other farmers to be fair; 2 = *Homo economicus* (selfish): keeps everything and does not trust other farmers to be fair; 3 = mutually beneficial joint maximizer: risk taking in investing everything in unknown farmers who they trust to be fair; 4 = self-oriented mutual benefit: cautiously invests a small amount, keeping most of the possible investment from the other farmer who they do not trust to be fair as much as the other 2 investing classes.

Farmers split into 4 latent classes to describe their investment and generosity decisions (Figure 1), using the complete responses from 417 farmers. An ellipsoidal, equal volume, and equal shape mixture model had the best BIC (BIC = 412.67, log-likelihood = 296.83, normalized entropy = 0.98). The 4 classes are described in detail in Supplemental File S3 (https://rdmc.nottingham.ac.uk/handle/internal/9483). Briefly, farmers in the first and largest class, “generous self-oriented mutual benefit,” invested half, expected an equal split of the investment back, and were generous (35% of farmers). This class was the most altruistic in the dictator game, but not willing to invest everything in farmers that they did not completely trust, so kept half of the possible investment. This investment game strategy means that farmers benefit overall but the investing farmer benefits the most. The second largest class, “*Homo economicus* (selfish),” invested nothing and kept everything (30% of farmers); these farmers could be classed as selfish profit maximizers who do not trust other farmers to be fair. The third largest class, “mutually beneficial joint maximizer,” invested everything, expected an equal split of the investment back, and gave some away (19% of farmers). This investment strategy maximizes the number of resources available to farmers in general, and therefore farming. This class of farmer would end up with the most total resources if the investment recipient returns the proportion of investment that the farmer expects. This strategy reveals a high level of trust in other farmers and a willingness to risk investing everything in other farmers. The final and smallest class, “self-oriented mutual benefit,” invested some, expected a less than equal split of the investment back, and gave very little away (16% of farmers). This class is less altruistic than the “generous self-oriented mutual benefit” class and invested less. These farmers are cautiously investing only a small amount to farmers who they do not trust to be very fair.

### Farmer Psychological Proximity to Others

The farmers felt closer to their cows (mean score of 6.0, Supplemental Figure S1) than any of the groups of people and were closer to known groups than unknown groups of people. Similar to their level of trust, the farmers felt closer to their veterinarian (4.8) than to neighboring farmers (3.9), the veterinary community (3.5), dairy farmers (3.4), beef farmers (3.4), the farming community (3.3), the National Farmers' Union (3.0), and the government (2.0). There was a range of scores of 1 to 7 for all groups.

### BVD Control Methods Used by Farmers

Farmers split into 9 latent classes to describe their BVD control decisions using the complete responses from 467 farmers; detailed descriptions of each class are given in Table 2. A spherical, equal volume mixture model had the best BIC (BIC = −5,269.64, log-likelihood = −2,330.58, entropy = 0.89). Farmers were fairly evenly split across all 9 classes: “doing nothing” (12%), “closed, separate and testing” (15%), “vaccinating” (17%), “vaccinating, testing and culling” (8%), “careful introducing new stock and testing” (9%), “careful introducing new stock, separate, and testing” (7%), “careful introducing new stock, separate, testing, and disinfecting people” (8%), “careful introducing new stock, separate, vaccinating, and testing” (14%), “careful introducing new stock, separate, vaccinating, testing, and disinfecting people” (11%).

**Table 2:** The percentage of 467 farmers in each of the 9 latent classes describing the farmer's approach to control of bovine viral diarrhea (BVD) and a description of the control measures undertaken by each class (with the percentage of farmers using a control measure in parentheses)^1^

Class	Percent	Typical practice (>60% of farmers)	Frequent practice (40–60% of farmers)	Atypical practice (<40% of farmers)
Doing nothing	12			Isolate or test new cattle (34%) Closed (25%) Milk test (13%) Buy only from BVD-free herds (8%) Separate from neighboring stock (8%) Blood or tissue test (8%) Cull PI (7%) Disinfect people (5%) Vaccinate (4%)
Closed, separate, and testing	15	Closed (99%) Separate (84%) Blood or tissue test (77%)	Disinfect people (58%) Milk test (57%) Vaccinate (51%)	Cull PI (9%)
Vaccinating	17	Vaccinate (98%)	Blood or tissue test (55%) Milk test (42%)	Closed (33%) Isolate or test new cattle (31%) Buy only from BVD-free herds (17%) Disinfect people (6%) Separate from neighboring stock (5%) Cull PI (2%)
Vaccinating, testing, and culling	8	Cull PI (98%) Blood or tissue test (88%) Vaccinate (86%) Milk test (66%) Isolate or test new cattle (63%)		Only buy from BVD-free herds (33%) Separate from neighboring stock (22%) Closed (17%) Disinfect people (13%)
Careful introducing new stock and testing	9	Isolate or test new cattle (81%) Blood or tissue test (88%) Buy only from BVD-free herds (70%)		Closed (15%) Milk test (7%) Vaccinate (6%) Disinfect people (3%) Separate from neighboring stock (1%)
Careful introducing new stock, separate, and testing	7	Separate from neighboring stock (99%) Blood or tissue test (87%) Isolate or test new cattle (84%) Buy only from BVD-free herds (62%)		Closed (23%) Cull PI (12%) Milk test (2%) Vaccinate (1%) Disinfect people (1%)
Careful introducing new stock, separate, testing, and disinfecting people	8	Isolate or test new cattle (100%) Disinfect people (99%) Buy from BVD-free herds (89%) Blood or tissue test (86%) Separate from neighboring stock (64%)		Closed (14%) Milk test (9%) Cull PI (9%) Vaccinate (2%)
Careful introducing new stock, separate, vaccinating, and testing	14	Vaccinate (99%) Test or isolate new stock (92%) Blood or tissue test (89%) Buy from BVD-free herds (88%) Separate from neighboring stock (84%)		Cull PI (17%) Milk test (16%) Closed (8%) Disinfect people (1%)
Careful introducing new stock, separate, vaccinating, testing, and disinfecting people	9	Disinfect people (99%) Vaccinate (98%) Test or isolate new stock (95%) Blood or tissue test (92%) Separate from neighboring stock (87%) Buy only from BVD-free herds (86%)		Cull PI (26%) Milk test (20%)

1 PI = persistently infected.

### Multinomial Logistic Regression of Disease Prevention

Latent classes that contained similar BVD management strategies were merged to reduce the number of classes in the multinomial model, resulting in 5 classes (Table 3). Briefly, these classes were (1) doing nothing, (2) closed herd, separated from neighboring stock and testing, (3) vaccinating and testing, (4) careful introducing new stock and testing, and (5) undertaking many prevention strategies with an open herd. Univariable model results examining demographic variables and psychological constructs are provided in Supplemental Tables S2 and S3 (https://rdmc.nottingham.ac.uk/handle/internal/9483), and the multivariable model results are presented in Table 4.

**Table 3:** The percentage of 467 farmers in each of the 5 latent classes describing the farmer's approach to control of bovine viral diarrhea (BVD) and a description of the control measures undertaken by each class (with the percent of farmers using a control measure in parentheses)^1^

Class	Percent	Typical practice (>60% of farmers)	Frequent practice (40–60% of farmers)	Atypical practice (<40% of farmers)
Doing nothing	12			Isolate or test new cattle (34%) Closed (25%) Milk test (13%) Buy only from BVD-free herds (8%) Separate from neighboring stock (8%) Blood or tissue test (8%) Cull PI (7%) Disinfect people (5%) Vaccinate (4%)
Closed, separate and testing	15	Closed (99%) Separate from neighboring stock (84%) Blood or tissue test (77%)	Disinfect people (58%) Milk test (57%) Vaccinate (51%)	Cull PI (9%)
Vaccinating and testing	25	Vaccinate (94%) Blood or tissue test (66%)	Milk test (50%) Isolate or test new cattle (41%)	Cull PI (33%) Closed (28%) Buy only from BVD-free herds (22%) Separate from neighboring stock (10%) Disinfect people (8%)
Careful introducing new stock and testing	16	Blood or tissue test (88%) Isolate or test new cattle (82%) Buy only from BVD-free herds (67%)	Separate from neighboring stock (44%)	Closed (19%) Cull PI (15%) Milk test (5%) Vaccinate (4%) Disinfect people (2%)
Careful introducing new stock, separate, vaccinating and testing	31	Test or isolate new cattle (95%) Blood or tissue test (89%) Buy only from BVD-free herds (88%) Separate from neighboring stock (80%) Vaccinate (74%)	Disinfect people (55%)	Cull PI (18%) Milk test (15%) Closed (11%)

1 PI = persistently infected.

**Table 4:** The results of a multivariable multinomial model of 380 UK cattle farmers in 5 bovine viral diarrhea (BVD) control classes explained by farmer psychosocial attitudes and capability, opportunity, motivation-behavior (COM-B) factors^1^

Reference BVD behavior class = doing nothing	Closed, separate, and testing			Vaccinating and testing			Careful introducing new stock and testing			Careful introducing new stock, separate, vaccinating, and testing		
	OR	95% CI	*P*-value	OR	95% CI	*P*-value	OR	95% CI	*P*-value	OR	95% CI	*P*-value
Generous self-oriented mutual benefit Ref = *Homo economicus* (selfish)	1.17	0.38–3.58	0.787	1.22	0.47–3.17	0.683	0.83	0.30–2.31	0.728	1.77	0.68–4.56	0.239
Mutually beneficial joint maximizer Ref = *Homo economicus* (selfish)	2.75	0.54–14.09	0.226	2.16	0.46–10.15	0.331	4.72	1.05–21.15	0.043	3.76	0.83–16.97	0.085
Self-oriented, mutual benefit Ref = *Homo economicus* (selfish)	2.28	0.49–10.63	0.293	2.34	0.61–9.01	0.217	0.94	0.21–4.22	0.930	3.64	0.97–13.63	0.055
Controlling disease for self and others	2.73	1.54–4.81	0.001	2.03	1.31–3.14	0.001	1.48	1.00–2.20	0.050	2.55	1.65–3.92	<0.001
Reflective motivation {0.57}	2.71	0.96–7.67	0.061	3.27	1.32–8.09	0.011	2.03	0.79–5.18	0.141	4.89	2.00–11.98	0.001
Automatic motivation {0.50}	1.93	0.74–5.00	0.177	2.77	1.16–6.61	0.021	2.02	0.83–4.94	0.124	3.95	1.69–9.26	0.002
Trust in farmers	0.51	0.28–0.91	0.024	0.79	0.46–1.36	0.392	0.84	0.49–1.45	0.533	0.52	0.31–0.89	0.016
Psychological proximity to your veterinarian	1.28	0.94–1.75	0.118	1.25	0.95–1.65	0.114	1.06	0.79–1.40	0.709	1.32	1.01–1.74	0.041
Psychological proximity to dairy farmers	2.26	1.39–3.67	0.001	2.03	1.30–3.17	0.002	0.91	0.58–1.43	0.688	1.08	0.71–1.63	0.727
Psychological proximity to beef farmers	0.37	0.22–0.62	<0.001	0.45	0.28–0.71	0.001	1.05	0.66–1.68	0.834	0.84	0.54–1.30	0.432
Psychological capability	5.65	1.99–16.05	0.001	1.50	0.61–3.70	0.383	1.30	0.52–3.26	0.572	2.08	0.86–5.00	0.102
Reflective motivation {0.55}	2.32	0.81–6.68	0.117	1.96	0.79–4.85	0.146	1.50	0.61–3.73	0.378	2.85	1.16–6.98	0.022
Automatic motivation {0.49}	1.65	0.61–4.47	0.321	1.82	0.75–4.40	0.182	1.51	0.63–3.65	0.357	2.55	1.07–6.03	0.034
Physical opportunity {0.32}	1.86	1.01–3.44	0.046	1.18	0.69–2.03	0.540	1.33	0.75–2.34	0.324	1.33	0.78–2.27	0.287

1 Variables correlated with variables in the model (correlation coefficient in brackets {}) and that would be included (*P* < 0.05) with the omission of the correlated factor are indented in the table and presented as alternatives below the correlated variable. Ref = reference economic games latent class; all other categories are on a continuous scale. OR = odds ratio.

In the multivariable model of 380 farmers, farmers who “controlled disease for themselves and others” were more likely to be in any class for BVD control than doing nothing. Farmers who did not trust other farmers, had high psychological capability, and had high physical opportunity were more likely to have a closed, separate herd and be testing than doing nothing. Farmers who did not trust other farmers were also more likely to be undertaking many prevention strategies with an open herd. Farmers with high automatic and reflective motivation were more likely to be in the classes that were vaccinating and testing, alone or in combination with other controls. Farmers with high psychological proximity to their veterinarian were more likely to be undertaking many prevention strategies in an open herd. Farmers with high psychological proximity to dairy farmers and low psychological proximity to beef farmers were more likely to keep their herd closed and separate and test or vaccinate and test than do nothing. Finally, farmers who were in the “mutually beneficial joint maximizers” latent class rather than selfish were more likely to be careful introducing new stock and testing rather than doing nothing.

There was no evidence of confounding of the demographic variables and little evidence of spatial autocorrelation (Supplemental File S2). There was no evidence of poor model fit in a Hosmer-Lemeshow goodness-of-fit test (χ^2^ = 25.9, df = 32, *P* = 0.768) or when evaluating cross-validated predictions.

## DISCUSSION

This study aimed to investigate farmer psychosocial factors and their association with the undertaking of on-farm BVD control measures. A key strength of the study was that we used established psychosocial measures and theory to underpin our survey questions and analysis. The major findings were that psychological proximity to veterinarians, trust in farmers, automatic and reflective motivation, psychological capability, and physical opportunity were important for farmer behavior regarding BVD control strategies; these are discussed in turn below.

Psychological proximity to veterinarians is a novel psychosocial construct of importance for cattle infectious disease control. Farmers who felt close to their veterinarian were more likely to do more to prevent and control BVD in an open herd. This is a novel field of application for the IOS scale and there is only limited research into psychological proximity in human health behavior (Bobak and Raupach, 2018; Tu et al., 2021). The IOS scale correlates with both feeling close and behaving close (Aron et al., 1992), which could explain why farmers who had higher psychological proximity to their veterinarian were more likely to do more to prevent and control BVD if they had an open herd, behaviors that tend to align with veterinary advice. Trust is part of the interpretation that respondents give to the IOS scale and the two are correlated (Kong, 2018; Kleinert et al., 2020). Therefore, farmers who felt closer to their veterinarian also had greater trust in veterinarians (correlation coefficient = 0.52). However, psychological proximity includes aspects other than just trust: behavioral closeness, connection with the other, independence from the other, and similarities with the other, which is also part of how respondents interpret the IOS scale (Aron et al., 1992). Trust in veterinarians is commonly found as an important factor in cattle farmer infectious disease control behavior in the literature (e.g., Brennan and Christley, 2013; Hernández-Jover et al., 2016; Bard et al., 2019); however, here psychological proximity was more important. Veterinarians and farmers frame biosecurity in different ways, hindering the veterinarian-farmer relationship (Shortall et al., 2016). Therefore, veterinarians have a role in increasing the psychological proximity that farmers feel with them and encouraging greater uptake of veterinary advice by paying attention to the broader aspects of psychological proximity than only trust, such as taking up a farmer perspective on disease control.

In contrast to the relationship with veterinarians, trust in farmers was more important than psychological proximity for BVD control, with a lack of trust in farmers associated with farmers either maintaining a closed herd separated from neighboring stock or using many methods of control if they had an open herd. Farmers often ensure that they buy animals from other farmers that they trust (Hernández-Jover et al., 2016), and maintaining a closed, separate herd offers even more protection against disease that may be transmitted from farmers that are not trusted. Lack of trust in other farmers has previously been reported as a barrier to biosecurity uptake, with farmers unwilling to contribute to collective action that they do not trust other farmers to engage in (Heffernan et al., 2008; Shortall et al., 2016). This leads them to support greater regulation by government to ensure that all farmers play their part in controlling disease (Heffernan et al., 2016). This opinion was also voiced by some of the farmers in the focus groups in this study in the context of national BVD eradication. In contrast, farmers who were prepared to invest everything in another unknown farmer in the economic games with a high expectation that the other farmer will be fair in return, the “mutually beneficial joint maximizers” class, were more likely to take care introducing new stock and test their cattle, than do nothing. High investment in the investment game indicates both a high level of trust in the other farmer and a willingness to take risks (Chetty et al., 2021). Maximizing the growth of the investment also leads to the greatest resource to the farming community, regardless of whether the other farmer returns any of the investment to the donor farmer. These community-minded, trusting farmers were more likely to rely on testing and the status of the herds that they buy from rather than be in the most self-protective behavior classes. Testing and checking herd status are both behaviors associated with BVD accreditation schemes, so these farmers may have bought into the importance of BVD control for the whole farming community. Most of the farmers surveyed invested less than the 50% that is typical in other research (Johnson and Mislin, 2011); therefore, although lack of trust in farmers is positive in terms of farmers taking responsibility for protecting their own herd from BVD, the tendency of farmers to think only of their own herd has implications for being able to achieve national disease control via voluntary and cohesive farmer action.

In terms of general other-regarding preferences, 75.4% of farmers were categorized as prosocial, with almost everyone else classed as individualistic. This distribution of SVO categories was similar to other studies but with a higher proportion of prosocials than the typical 65% (Murphy and Ackermann, 2014). Prosocials can be further divided into those who wish to minimize inequality between themselves and someone else and those who wish to maximize joint gain. Differentiating between these groups requires additional SVO slider measure questions (Murphy et al., 2011), which were not included in the survey to maintain brevity and achieve a good response rate. Further research into how prosocial UK farmers subdivide and any associations with infectious disease control behavior would be worthwhile. When exploring broader other-regarding preferences (i.e., altruism, prosociality, and trust) in the dictator and trust games we identified 4 classes of other-regarding preferences for farmers. Therefore, farmers are heterogeneous in terms of the other-regarding strategies and these differences need to be accounted for to understand how farmers' other-regarding preferences influence behavior.

Farmers with high motivation to control infectious diseases were more likely to use vaccination, especially with other preventive measures. Both reflective (goals and decision making) and automatic (habits and emotions) motivation were associated with vaccine use, with a slightly higher effect from reflective motivation. Aspects of reflective motivation have been well researched in infectious disease control and farmers are more likely to control disease in their herd if they take responsibility for disease control, want to see the benefits of controlling infectious diseases, or have goals to reduce or remove disease from their herd (Ellis-Iversen et al., 2010; Azbel-Jackson et al., 2018; Robinson, 2020). The emotional aspects of automatic motivation are known to affect farmer behavior (O'Kane et al., 2017). Infectious disease is frequently an emotive subject for farmers and worry often leads farmers to take preventive action to prevent the negative consequences of disease (Suit-B et al., 2020; Doidge et al., 2021). Habit could also be a factor and habitually getting vaccinated is important in human vaccination behavior (Pot et al., 2017). Habit is often a barrier to changing farmer behavior (Coyne et al., 2020) and some farmers in the focus groups viewed the BVD vaccine as insurance. Vaccination could therefore be being used both habitually once a farmer starts vaccinating and as a protection against the worry of a BVD outbreak. Both motivation factors were correlated with the “controlling disease for self and other” factor, which is unsurprising because motivation is closely associated with behavior in the COM-B framework and farmers who were proactively controlling disease would have scored highly in the “controlling disease for self and other” factor (Michie et al., 2011).

Farmers who felt that they understood how and why they should control infectious disease (psychological capability) and had the time and money to do so (physical opportunity) were more likely to keep a closed herd, separated from neighboring stock, and undertake BVD testing. Although maintaining a closed, separate herd is very effective at preventing many infectious diseases, it is not very practical for farmers (Shortall et al., 2017). Psychological capability and physical opportunity were correlated with each other and with automatic and reflective motivation and form the context for farmer behavior; therefore, they all need to be taken into account when considering behavior change (Michie et al., 2011). Psychological capability had a greater effect on behavior than physical opportunity, suggesting that knowledge and understanding of how and why to prevent disease is more important for maintaining a closed and separate herd than time or money, perhaps because farmers often appreciate the economic benefits of preventing and controlling disease in their cattle (Oliveira et al., 2018; Robinson, 2020). There has been substantial research into how to encourage farmers to uptake disease control behavior and this finding highlights the importance of effective knowledge transfer to farmers, but this needs to be in combination with ensuring that farmers have the physical resources to carry out the behavior.

Our findings are likely to be generalizable to other similar endemic diseases and farmers in other countries with similar experiences of veterinarians, government, and neighbors; however, further study will be needed to investigate this. The farmers in the study were biased toward English farmers (73% were English compared with 48% in the national population; Agriculture and Horticulture Development Board, 2019), therefore, the psychosocial profiles of the general cattle farmer population may be slightly different. However, country was not a confounder in the multinomial model. Respondents may also have been more interested in infectious diseases than the general farming population; however, 12% of farmers were still doing very little if anything to control BVD and differences were found in both farmer attitudes and BVD control practices.

From these findings, we recommend that trust and proximity to veterinarians and farmers may be crucial to enhancing infection control. One way to achieve this may be to capitalize on conditional cooperation effects (Fischbacher et al., 2001). Conditional cooperation occurs when people are aware that others are also cooperating, and this increases the probability that they will cooperate (Rustagi et al., 2010). This requires making others' cooperation behavior observable (Bradley et al., 2018) and has been effectively implemented using social media (Cameron et al., 2013).

In conclusion, psychosocial factors are important for UK cattle farmer uptake of BVD control. Psychological proximity to veterinarians was a novel factor associated with proactive BVD control and was more important here than the more extensively investigated trust. In addition, lack of trust in other farmers, a high understanding of how and why to control infectious disease, time and money, and both automatic and reflective motivation were also associated with farmers' approach to BVD control. These findings highlight the importance of a close veterinarian-farmer relationship and are important for promoting effective BVD control by farmers, which has implications for successful nationwide BVD control and eradication schemes.
